# A Case of Endometrioid Adenocarcinoma Arising from Adenomyosis

**DOI:** 10.1155/2014/569295

**Published:** 2014-02-10

**Authors:** Shigeki Taga, Mari Sawada, Aya Nagai, Dan Yamamoto, Ryoji Hayase

**Affiliations:** Department of Obstetrics and Gynecology, National Hospital Organization Fukuyama Medical Center, Okinogamicho 4-14-17, Fukuyama 720-0825, Hiroshima Prefecture, Japan

## Abstract

Malignant changes in endometriosis are often reported, but those in adenomyosis are rare. We report a case of endometrioid adenocarcinoma arising from adenomyosis. *Case Presentation*. A 57-year-old woman presenting with vaginal bleeding was referred to our hospital. Cytological tests of endometrium revealed atypical glandular cells. Fractional endometrial curettage revealed normal endometrium without atypia. Magnetic resonance imaging (MRI) revealed multiple myomas. The endometrium was slightly enhanced on T_1_-weighted imaging and endometrial cancer was suspected. Myometrial invasion was not evident. The patient was admitted and semiradical hysterectomy with bilateral salpingo-oophorectomy and pelvic lymphadenectomy was performed. Histopathological study revealed grade 1 endometrioid adenocarcinoma. Although the lesion was located in the muscle layer of the corpus and invaded more than half of it, the endometrium was intact. Pelvic lymph node metastasis was noticed. No cervical invasion or metastasis to the adnexa was seen. We diagnosed the case with a stage 1B endometrioid adenocarcinoma originating from adenomyosis. Adjuvant chemotherapy was then performed in the form of 5 cycles of paclitaxel (180 mg/m^2^) and carboplatin (AUC = 5). Five years later, right lung metastasis and right para-aortic and pelvic lymph nodes metastasis were noticed. Paclitaxel and carboplatin are now being administered.

## 1. Introduction

Endometrial cancer arising from endometriosis has often been reported recently. However, malignant tumors arising from adenomyosis are rare. Endometrial cytology usually fails to reveal malignant cells, and diagnosis is often delayed. Herein we report a case of endometrioid adenocarcinoma arising from adenomyosis and review the literature.

## 2. Case Report

**Figure 1:**
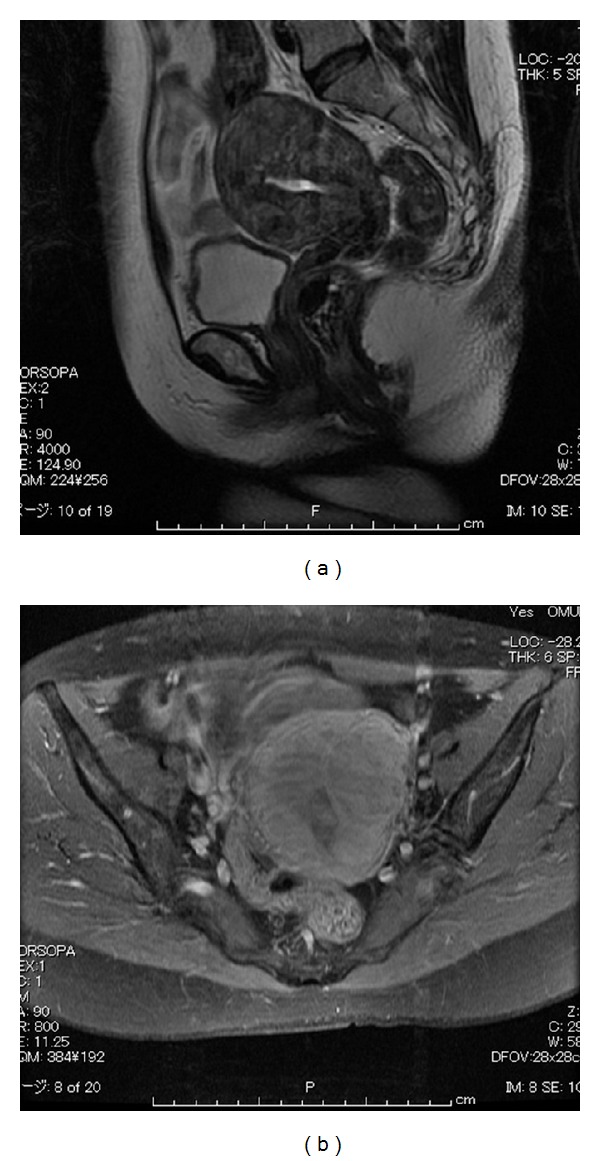
Magnetic resonance imaging revealed multiple myomas. The endometrium was enhanced on T_1_-weighted imaging and endometrial cancer was suspected (b). Myometrial invasion was not evident.

A 57-year-old postmenopausal woman, gravida 4, para 3, presenting with vaginal bleeding visited a local clinic. Cytological tests of endometrium and endocervix revealed suspected malignant cells. She was referred to our hospital. Cytological tests of endometrium revealed atypical glandular cells. Fractional endometrial curettage revealed normal endometrium without atypia. Ultrasound scans revealed a slightly thickened endometrium. Magnetic resonance imaging (MRI) revealed multiple myomas. The endometrium was enhanced on T_1_-weighted imaging and endometrial cancer was suspected. Myometrial invasion was not evident (Figure 1).

**Figure 2:**
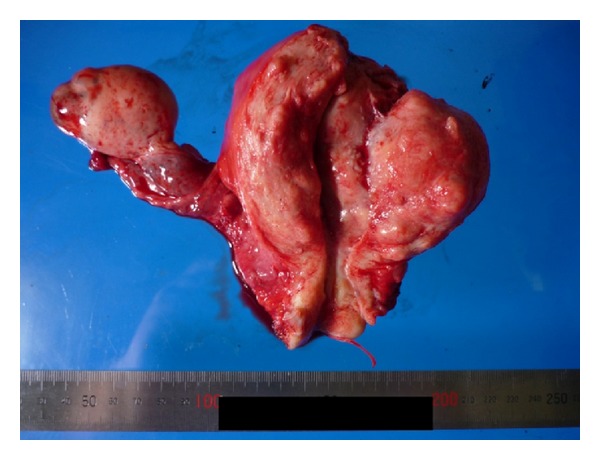
The surgical specimen. The endometrium appeared normal on gross examination.

Serum levels of CA125 and CA19-9 were elevated to 617.3 U/mL and 134.6 U/mL, respectively. The patient was admitted and semiradical hysterectomy with bilateral salpingo-oophorectomy and pelvic lymphadenectomy was performed. There was no peritoneal dissemination or ascites. The uterus was normal sized and the right ovary was somewhat enlarged and looked like a mature cystic teratoma (Figure 2).

**Figure 3:**
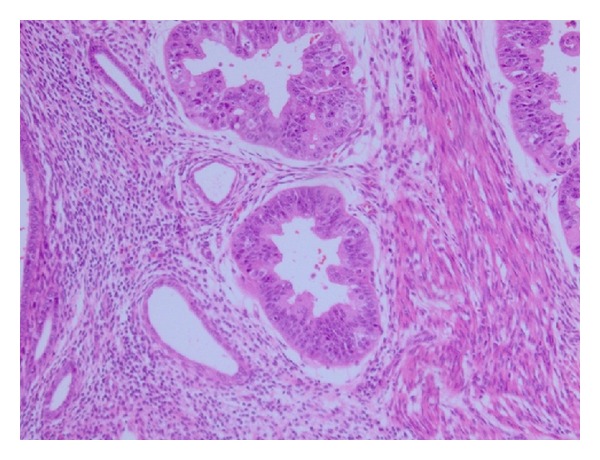
(H.E. ×100). Endometrioid adenocarcinoma adjacent to the adenomyotic foci was observed.

Histopathological study revealed grade 1 endometrioid adenocarcinoma. However, the lesion was located only in the muscle layer of the corpus and the endometrium was intact. Cancer nests adjacent to the f adenomyotic foci were observed (Figure 3).

Left iliac lymph node metastasis was confirmed. According to the revised staging of the international Federation of Gynecology and Obstetrics (FIGO) we diagnosed the case with a stage 1B endometrioid adenocarcinoma originating from adenomyosis. Peritoneal cytology was negative. Postoperative classification was pT1BN1M0. A mature cystic teratoma of the left ovary was confirmed. There were adenomyosis and leiomyomas in the corpus muscle layer. No cervical invasion or metastasis to the adnexa was seen. Adjuvant chemotherapy was then performed in the form of 5 cycles of paclitaxel (180 mg/m^2^) and carboplatin (AUC = 5). Five years later, right lung metastasis and right para-aortic and pelvic lymph nodes metastasis were noticed. Paclitaxel and carboplatin are now being administered.

## 3. Discussion

Endometrial cancer is the most common gynecological tumor [1, 2]. Endometrial cancer arising from endometriosis has often been reported recently. However, adenocarcinoma arising from adenomyosis is a rare entity. Koshiyama et al. reported only four cases in 564 patients (0.74%) operated between 1981 and 2001 [3]. This entity should be distinguished from extension of endometrial adenocarcinomas arising from the eutopic endometrium to the adenomyosis. When an endometrial carcinoma and adenomyosis coexist in the same uterus, adenomyosis is invaded by the carcinoma in approximately 25% of the cases. Colman et al. modified the diagnostic criteria proposed by Sampson for ovarian cancer arising from endometriosis so as to apply the criteria to carcinomas arising from adenomyosis as follows [4, 5]. (a) The carcinoma must not be situated in the endometrium or elsewhere in the pelvis; (b) the carcinoma must be seen to arise from the epithelium of adenomyosis and not to have invaded from other source; and (c) endometrial (adenomyotic) stromal cells should be surrounding the aberrant glands to support the diagnosis of adenomyosis.

There are two pathways of carcinogenesis of this condition. One is de novo malignant transformation inside adenomyotic foci while the eutopic endometrium was unaffected. Three of 4 cases that Koshiyama et al. reported (a grade 3 endometrioid carcinoma, a clear cell adenocarcinoma, and a serous papillary adenocarcinoma) were histologically unfavorable subtype. They assumed this might contribute to a poor prognosis. Another is simultaneous malignant changes of the eutopic endometrium and adenomyosis. Kucera et al. analysed the clinical data of 219 patients with the diagnosis of early endometrial cancer and reported that malignant changes in adenomyosis were present in 6.8% of patients with endometrial cancer. All those 6 cases were with endometrioid adenocarcinoma. Five cases were well or moderately differentiated. And they suggested that there is a similar pathway of carcinogenesis in adenomyosis as is known in estrogen-responsive endometrial cancer type 1 [6].

Diagnosis is often delayed because of the absence of lesion in eutopic endometrium. Boes et al. reported a case which was diagnosed 1 year after the first symptom. In their case, hysteroscopy was negative and hormonal treatment was continued. At last, hysteroscopic excision of the endometrial polyp revealed adenocarcinoma. According to their review, in almost all the cases where endometrial cytology was performed, cytology was negative [2]. Diagnosis is made usually when the tumor has grown to involve the endometrium, causing abnormal uterine bleeding or has spread outside of the uterus [2]. Motohara et al. reported that they observed a patient every 6 months after being diagnosed with adenomyosis; eleven years after the first diagnosis, endometrial cytology revealed malignant cells and MRI demonstrated replacement of the adenomyotic lesion by a poorly demarcated lesion. The management of this clinical entity consists of surgery and chemotherapy.

In conclusion, adenocarcinoma arising from adenomyosis is a rare entity, and diagnosis is usually difficult and often delayed. This clinical entity should be kept in mind especially when an adenomyosis patient is followed for a long period.
